# Factors Associated With Unintended Dural Puncture and Failed Neuraxial Anesthesia in Obstetric Anesthesia

**DOI:** 10.7759/cureus.107705

**Published:** 2026-04-25

**Authors:** Manuel C Vallejo, Christa L Lilly, Ayten Saracoglu, Kemal T Saracoglu, Hiram Acevedo Bonilla, Mongan D Paul, Kubra Cakar Yilmaz, James G Cain

**Affiliations:** 1 Anesthesiology, University of Florida College of Medicine - Jacksonville, Jacksonville, USA; 2 Epidemiology and Biostatistics, West Virginia University, Morgantown, USA; 3 Obstetrics and Gynecology, Diyarbakır Gazi Yaşargil Training and Research Hospital, University of Health Sciences, Diyarbakir, TUR

**Keywords:** dural puncture, failed epidural, failed spinal anesthesia, labor analgesia, maternal outcome, quality improvement initiative, risk exposure, risk factors‎

## Abstract

Objective: Unintended dural puncture (UDP) and failed neuraxial anesthesia increase risks for conversion to general anesthesia, procedural delays, and patient dissatisfaction. Additionally, UDP can result in a postdural puncture headache (PDPH) with significant maternal morbidity. Factors such as clinician experience, multiple attempts, anatomical variation, and technical difficulty are modifiable areas for quality improvement. We aim to identify specific factors associated with UDP and failed regional at our institution.

Methods: A one-year (July 2024-July 2025) retrospective electronic medical record-deidentified cohort study was conducted as a quality-improvement registered project. Controls (n = 126) were compared with UDP (n = 41) and failed neuraxial anesthesia (n = 38). Factors analyzed included demographic data (age, height, weight, BMI, gravidity, parity, and gestation) American Society of Anesthesiologists physical status classification, trial of labor, anesthesia type (spinal, epidural, combined spinal-epidural (CSE), dural puncture epidural, and general), intrathecal catheter placement, conversion to general anesthesia, preeclampsia diagnosis, block attempts, provider status (resident, certified registered nurse anesthetist (CRNA), and attending), failed regional anesthetic, and PDPH. Interval data were analyzed using the t-test; nominal data were analyzed using the chi-square test, including odds ratios. Assumption corrections included Mann-Whitney nonparametric testing, unequal variance corrections on t-tests, and Fisher’s exact tests, as needed. Alpha was set to 0.05.

Results: Over this one-year period, 1,429 obstetrical cases were performed, with a UDP rate of 41 (2.9%) and a failed regional rate of 38 (2.7%). Factors significant for UDP included age, CSE anesthesia technique, provider status (more likely for a resident than a CRNA), more block attempts, intrathecal catheter placement, preeclampsia diagnosis, and PDPH.

Factors significant for failed regional included older age, younger gestation, trial of labor, spinal anesthesia technique more likely than epidural, more block attempts, cesarean delivery, general anesthesia conversion, and preeclampsia diagnosis.

Conclusion: Our analysis identifies older maternal age, preeclampsia, multiple block attempts, and provider experience as key risk factors for UDP and failed regional, highlighting modifiable residency training targets to improve obstetric anesthetic outcomes.

## Introduction

Neuraxial anesthesia (e.g., spinal, epidural, dural puncture epidural (DPE), and combined spinal-epidural (CSE)) is advocated over intravenous and general anesthesia for labor analgesia and cesarean delivery (CD) due to its favorable maternal and neonatal safety profile [1]. Neuraxial complications include unintended dural puncture (UDP) and postdural puncture headache (PDPH) [2]. Despite widespread use, failure rates for spinal anesthesia range from 1% to 9% [1-4], while epidural failure rates range from 6% to 30%, often necessitating conversion to general anesthesia and contributing to patient dissatisfaction and increased perioperative risk [1,5-7]. In addition, a UDP occurs in approximately 0.5%-6% of obstetric epidural placements and is frequently complicated by PDPH, which may result in prolonged hospitalization, impaired maternal-infant bonding, and need for invasive interventions such as epidural blood patch [2,8-10].

Several factors have been associated with UDP and failed neuraxial anesthesia, including clinician experience, patient anatomy, urgency of delivery, and technical difficulty requiring multiple attempts [1,11]. Many of these factors are potentially modifiable through focused education, supervision, and system-level interventions. The Accreditation Council for Graduate Medical Education emphasizes quality improvement and patient safety as core competencies, making obstetric anesthesia an ideal setting to identify training-related opportunities for improvement [12].

We aimed to identify patient-, procedural-, and provider-related factors associated with UDP and failed neuraxial anesthesia at our institution. A secondary aim was to identify actionable targets for anesthesia residency quality improvement in labor and delivery.

## Materials and methods

Study design and setting

This study was conducted as a retrospective cohort, deidentified, quality improvement registered project (QIPR) at a tertiary academic medical center. QIPR approval for the study was obtained. Institutional review board oversight was waived in accordance with institutional policy for quality improvement initiatives.

Study population and data sources

All electronic medical record (EMR) obstetric anesthetic cases performed between July 2024 and July 2025 were collected, screened, and deidentified before data analysis. UDP cases (n = 41) and failed neuraxial anesthesia cases (n = 38) were identified and matched in a 3:1 ratio to controls (n = 126) without UDP or failed neuraxial anesthesia.

Exposure groups

UDP was defined as an unintended dural puncture occurring during epidural needle or catheter placement, resulting in return of cerebrospinal fluid (CSF). This is distinct from the intentional dural puncture performed during the spinal component of a CSE technique. When a UDP occurs, the initial procedure interspace is abandoned, and either a different lumbar vertebral interspace site is chosen for epidural catheter placement, or the epidural catheter is inserted intrathecally, depending on the anesthesiologist for patient management.

A failed neuraxial anesthetic was defined as inappropriate labor analgesia despite suitable dosing within the first 30 minutes of the procedure. Failed neuraxial for CD was defined as needing conversion to an alternative anesthetic technique for surgical delivery. If an emergent CD was necessary during labor, the anesthesia provider discontinued the epidural infusion and provided a catheter bolus for surgical analgesia. In the event the epidural catheter did not provide adequate surgical analgesia, general endotracheal anesthesia was performed.

Risk factors

Measured variables included risk factors such as demographic data (age, height, weight, and BMI), obstetric characteristics (gravidity, parity, gestational age, and trial of labor), comorbidities (American Society of Anesthesiologists physical status and preeclampsia), anesthetic variables (anesthesia type: spinal, epidural, CSE, DPE, and general anesthesia), number of block attempts, and provider status (resident, student nurse anesthetist, certified registered nurse anesthetist (CRNA), or attending anesthesiologist).

Outcomes

Several variables, including intrathecal catheter placement, PDPH, CD, and conversion to general anesthesia, were examined and represent downstream procedural or clinical outcomes rather than antecedent risk factors. PDPH is defined in this study as a headache that occurs as a complication following a neuraxial procedure, typically worsening with sitting or standing and improving or resolving when lying supine.

Data analysis

Analyses were completed in Statistical Analysis System 9.4 (SAS Inc., Cary, NC). Means and standard deviations were used to describe continuous variables; medians and interquartile ranges (IQRs) for nonnormal continuous variables; and frequencies and valid percentages for categorical data. Data were treated with pairwise deletion and alpha set to 0.05 unless otherwise specified. For all risk factor and clinical outcomes analyses, group differences (vs. control) were examined as the exposure variable, and student independent-samples t-tests (for continuous variables) and chi-square tests (for categorical variables) were conducted. Assumption violations included homogeneity of variance, so for some analyses, unequal-variance correction was applied to the t-test. Nonnormal continuous outcomes were also examined using Mann-Whitney nonparametric tests. Chi-square tests that violated the assumption of small cell sizes additionally had Fisher's exact test run. For 2-by-2 tables, odds ratios (ORs) are also calculated and reported.

## Results

During the study period, 1,429 obstetric anesthetic cases were performed. The incidence of UDP was 41 (2.9%), and the incidence of failed neuraxial anesthesia was 38 (2.7%). Of the original 1,429 cases, 205 participants (14.3%) were included in the analytic cohort, including 126 controls (9.3% of all control cases). All UDP cases (n = 41) and failed neuraxial anesthesia cases (n = 38) were included.

Unintended dural puncture

Among the analytic cohort, 41 patients experienced UDP and were compared with 126 patients without UDP (Table 1). Patients with UDP were significantly older than controls (mean age = 28.4 ± 6.9 vs. 24.3 ± 5.8 years, t = -3.75, p = 0.0002). Anesthesia technique differed between the groups: CSE anesthesia was more common among patients with UDP (13 (31.7%) vs. 17 (13.5%), OR = 2.98, 95% CI = 1.29-6.85; chi-square = 6.95, p = 0.0083). The median number of block attempts was higher in the UDP group (median 2 (IQR = 1-2) vs. 1 (IQR = 1-1); Mann-Whitney Z = 5.85, p < 0.0001). Procedures performed by residents were more frequent among UDP cases than controls (26 (63.4%) vs. 37 (29.4%), chi-square = 15.27, p < 0.0001). In contrast, procedures performed by CRNAs were less common among UDP cases (6 (14.6%) vs. 61 (48.4%); chi-square = 14.69, p = 0.0001).

**Table 1:** Risk factors and outcomes for unintentional dural puncture ^a^t-test, Mann-Whitney test, or chi-square test ^b^Mann-Whitney reported ^c^Exact p value reported UDP: unintended dural puncture; OR: odds ratio; CSE: combined spinal-epidural; CRNA: certified registered nurse anesthetist; SRNA: student registered nurse anesthetists; ITC: intrathecal catheter; PDPH: postdural puncture headache; n/a: not available

Complication	Unit or category	UDP (n = 41)	Control (n = 126)	Test value^a^	p value	OR	95% CI
Age	Years	28.4 ± 6.9	24.3 ± 5.8	-3.75	0.0002	n/a	n/a
Anesthesia type	CSE	13 (31.7%)	17 (13.5%)	6.95	0.0083	2.98	1.29-6.85
	Epidural	29 (70.7%)	96 (76.2%)	0.49	0.48	0.76	0.34-1.66
Block attempts	n^b^	2 (1-2)	1 (1-1)	5.85	<0.0001	n/a	n/a
Provider	Resident	26 (63.4%)	37 (29.4%)	15.27	<0.0001	4.17	1.98-8.76
	CRNA	6 (14.6%)	61 (48.4%)	14.69	0.0001	0.18	0.07-0.46
	SRNA	6 (14.6%)	23 (18.3%)	0.28	0.6	0.77	0.29-2.04
	Attending^c^	3 (7.3%)	5 (4.0%)	0.76	0.41	1.91	0.44-8.37
ITC placement	Y/N^c^	20 (48.8%)	0 (0%)	69.83	<0.0001	n/a	n/a
PDPH	Y/N^c^	5 (12.2%)	1 (0.8%)	11.61	0.0036	17.36	1.96-153.4
Preeclampsia	Y/N	11 (26.8%)	16 (12.7%)	4.56	0.03	2.52	1.06-6.0

Several outcomes differed between the groups. Intrathecal catheter placement occurred in nearly half of UDP cases 20 (48.8%) and in none of the control cases (chi-square = 69.83, p < 0.0001). PDPH was also more frequent among UDP cases (5 (12.2%) vs. 1 (0.8%); chi-square = 11.61, p = 0.0036). Preeclampsia was more prevalent among patients with UDP (11 (26.8%) vs. 16 (12.7%); OR = 2.52, 95% CI = 1.06-6.00; chi-square = 4.56, p = 0.03).

Failed neuraxial anesthesia

Thirty-eight patients with failed neuraxial anesthesia were compared with 126 controls (Table 2). Patients with failed neuraxial anesthesia were older (mean age = 28.1 ± 6.5 vs. 24.3 ± 5.8 years, t = -3.46, p = 0.0007) and had a younger mean gestational age at delivery (36.4 ± 5.0 vs. 38.3 ± 2.6 weeks, Mann-Whitney Z = -2.14, p = 0.0321). Patients with failed neuraxial anesthesia were less likely to undergo a trial of labor than controls (21 (55%) vs. 110 (88%); chi-square = 19.49, p < 0.0001). Epidural anesthesia was less common among failed neuraxial cases (22 (57.9%) vs. 96 (76.2%), chi-square = 4.82, p = 0.03). In contrast, spinal anesthesia was more frequently observed among failed neuraxial cases (13 (34.2%) vs. 5 (4.0%), chi-square = 27.33, p < 0.0001). Procedural difficulty was greater among failed neuraxial cases, with a higher median number of block attempts compared with controls (median 1 (IQR = 1-2) vs. 1 (IQR = 1-1); Mann-Whitney Z = 2.55, p = 0.0108).

**Table 2:** Risk factors and outcomes for failed neuraxial anesthesia ^a^t-test, Mann-Whitney test, or chi-square test ^b^Mann-Whitney reported ^c^Exact p value reported OR: odds ratio; CSE: combined spinal-epidural; GETA: general endotracheal anesthesia; n/a: not applicable

Complication	Unit or category	Failed neuraxial (n = 38)	Control (n = 126)	Test value^a^	p value	OR	95% CI
Age	Years	28.1 ± 6.5	24.3 ± 5.8	-3.46	0.0007	n/a	n/a
Gestation	Weeks^b^	36.4 ± 5.0	38.3 ± 2.6	-2.14	0.0321	n/a	n/a
Labor trial	Y/N	21 (55%)	110 (88%)	19.79	<0.0001	0.17	0.07-0.39
Anesthesia type	CSE	8 (21.0%)	17 (13.5%)	1.29	0.26	1.71	0.67-4.34
	Epidural	22 (57.9%)	96 (76.2%)	4.82	0.03	0.43	0.20-0.93
	Spinal	13 (34.2%)	5 (4.0%)	27.33	<0.0001	12.58	4.12-38.48
Block attempts	N^b^	1 (1-2)	1 (1-1)	2.55	0.0108	n/a	n/a
Cesarean section	Y/N	28 (73.7%)	26 (20.3%)	37.20	<0.0001	10.77	4.64-24.97
GETA conversion	Y/N^c^	25 (65.8%)	4 (3.2%)	78.64	<0.0001	58.65	17.66-194.82
Preeclampsia	Y/N	11 (28.9%)	16 (12.7%)	5.60	0.0179	2.8	1.17-6.72

Outcome differences included CD, which occurred more frequently among patients with failed neuraxial anesthesia (28 (73.7%) vs. 26 (20.3%); chi-square = 37.2, p < 0.0001). Conversion to general endotracheal anesthesia was also markedly more common among failed neuraxial cases (25 (65.8%) vs. 4 (3.2%); chi-square = 78.64, p < 0.0001). Preeclampsia was more frequent in patients with failed neuraxial anesthesia compared with controls (11 (28.9%) vs. 16 (12.7%); chi-square = 5.6, p = 0.0179).

## Discussion

This quality improvement analysis identified several overlapping risk factors for UDP and failed neuraxial anesthesia, including older maternal age, anesthesia technique, multiple block attempts, and preeclampsia. These findings are consistent with prior literature demonstrating increased technical difficulty and reduced neuraxial success in patients with altered spinal anatomy or hypertensive disorders of pregnancy [1,11,13,14]. In a prospective multicenter analysis of 1,214 patients looking at the incidence and risk factors for spinal failure, Fuzier et al. noted a spinal failure incidence of 3.2%, and found that three or more puncture attempts, use of supplemental intravenous medication, and age less than 70 were factors associated with spinal failure [4]. Technical issues, such as the inability to obtain CSF, are the most common explanations reported in the literature [4]. Our study identified puncture attempts and age, as noted by Fuzier et al. [4], to be risk factors for UDP. Furthermore, we identified anesthetic technique, provider status, and preeclampsia diagnosis as additional UDP predictors.

Failed neuraxial anesthesia was predicted by maternal age, younger gestational age, trial of labor, anesthesia technique, increased number of block attempts, CD, conversion to general anesthesia, and preeclampsia. The etiology of failed neuraxial anesthesia is probably multiplexed and associated with many factors [5]. Inadequate epidural block causes include incorrect initial placement, catheter migration, and inadequate dosing [6]. In 673 patients, Grap et al. [5] looked at failed labor epidural requiring general anesthesia conversion for CD and noted the rate of failed epidural was 21%, and risk factors were younger maternal age (p = 0.0002) and augmentation with either intravenous fentanyl (p < 0.0001) or midazolam (p = 0.0008) during CD. A higher risk of conversion failure was also associated with a more urgent CD [5]. General anesthesia in the pregnant patient increases the risk for difficult airway, apnea, and aspiration. Additionally, drugs used during anesthesia can cross the placenta and increase the need for neonatal resuscitation at delivery. In an urgent CD, there is less time available to redose the epidural catheter; hence, general anesthesia is the more reliable choice to ensure adequate anesthesia.

Provider status and number of block attempts are particularly notable modifiable factors associated with UDP. As in our study, prior studies have demonstrated higher UDP rates with trainee-performed epidurals, particularly during early training, underscoring the importance of graduated responsibility, direct supervision, and structured feedback [11,14]. Simulation-based training and ultrasound-assisted neuraxial techniques have been shown to further mitigate risk by improving technical proficiency and anatomical recognition [15-17]. As a result of this study, we plan to schedule an annual neuraxial resident training workshop that incorporates simulation-based and ultrasound training to improve proficiency.

The association between failed neuraxial anesthesia and trial of labor, CD, and conversion to general anesthesia highlights the downstream impact of inadequate neuraxial function on maternal safety. Given the increased risks associated with general anesthesia in obstetrics, strategies to optimize initial neuraxial success are essential [7,11].

Overall, good clinical training is key to success. Collectively, our data support targeted residency quality-improvement initiatives focused on procedural standardization, limiting repeated attempts, enhancing attending supervision during high-risk cases, and incorporating simulation and ultrasound training into obstetric anesthesia curricula [15-17].

Limitations

This retrospective, single-center study may not be generalizable to other health care settings. Residual confounding and EMR documentation variability are also possible. Despite these limitations, the findings provide multiple actionable insights into residency training and quality improvement.

## Conclusions

Older maternal age, preeclampsia, multiple block attempts, and provider experience were key risk factors associated with UDP and failed neuraxial anesthesia in obstetric patients. These findings highlight modifiable targets for anesthesia residency quality improvement and support focused educational and system-level interventions to improve obstetric anesthetic outcomes. More studies to further examine the causes of UDP and failed neuraxial anesthesia are warranted.
